# The future trajectory of adverse outcome pathways: a commentary

**DOI:** 10.1007/s00204-018-2183-2

**Published:** 2018-03-16

**Authors:** Fiona Sewell, Nichola Gellatly, Maria Beaumont, Natalie Burden, Richard Currie, Lolke de Haan, Thomas H. Hutchinson, Miriam Jacobs, Catherine Mahony, Ian Malcomber, Jyotigna Mehta, Graham Whale, Ian Kimber

**Affiliations:** 1 National Centre for the Replacement, Refinement and Reduction of Animals in Research (NC3Rs), Gibbs Building, 215 Euston Road, London, NW1 2BE UK; 2 GlaxoSmithKline, Stevenage, Hertfordshire UK; 3 Syngenta Ltd., Jealott’s Hill International Research Centre, Bracknell, Berkshire UK; 4 MedImmune, Granta Park, Cambridge, UK; 5 School of Geography, Earth and Environmental Sciences, University of Plymouth, Plymouth, UK; 6 Public Health England, Chilton, Oxfordshire UK; 7 Procter and Gamble Company, Egham, Surrey UK; 8 Unilever, Safety and Environmental Assurance Centre, Sharnbrook, Bedford, UK; 9 Dow AgroSciences, European Development Centre, Milton Park, Abingdon, Oxfordshire UK; 10 Shell, Health, Shell Centre, London, UK; 11 Faculty of Biology, Medicine and Health, University of Manchester, Manchester, UK

**Keywords:** Adverse outcome pathways (AOPs), Hazard identification, Safety assessment, Risk assessment

## Abstract

The advent of adverse outcome pathways (AOPs) has provided a new lexicon for description of mechanistic toxicology, and a renewed enthusiasm for exploring modes of action resulting in adverse health and environmental effects. In addition, AOPs have been used successfully as a framework for the design and development of non-animal approaches to toxicity testing. Although the value of AOPs is widely recognised, there remain challenges and opportunities associated with their use in practise. The purpose of this article is to consider specifically how the future trajectory of AOPs may provide a basis for addressing some of those challenges and opportunities.

## Introduction

Adverse outcome pathways (AOPs) are a relatively recent development in mammalian and environmental toxicology. In 2012 the Organisation for Economic Cooperation and Development (OECD) launched a programme for the development of AOPs as pragmatic analytical constructs for the description of linked causally related events and that result in the manifestation of adverse health or environmental effects. They are characterised by the identification of a molecular initiating event (MIE) and a series of key events (KE) that flow from the MIE that represent mandatory steps, usually of increasing biological complexity, in the pathway to elicitation of an adverse effect (Ankley et al. 2010; Vinken 2013; OECD 2013, 2018). One illustrative example of this approach is the development of an AOP for skin sensitisation; perhaps one of the most complete AOPs currently available (OECD 2012).

One of the benefits that derive from the AOP initiative is a renewed focus on the importance of understanding the mode of action (MOA) and exploiting that understanding to improve hazard characterisation and risk assessment. Moreover, AOPs have been of value in driving the development and application of non-animal approaches to hazard and risk assessment (Burden et al. 2015; Jacobs et al. 2016), and are now also being employed in the development of defined approaches for in vitro OECD test guidelines.

Despite the undoubted benefits that have been afforded by AOPs, there are nevertheless challenges associated with their application, and opportunities to extend further their contributions to toxicological science, and protecting human health and the environment (Hutchinson et al. 2013; Perkins et al. 2015; Oki et al. 2016; Wittwehr et al. 2017; Leist et al. 2017; LaLone et al. 2017). Against that background, the purpose of this article is to consider the future trajectory of AOPs and how new developments might allow challenges and opportunities to be addressed, including their more effective use in screening and risk assessment and their application in a regulatory context.

## The future trajectory of AOPs

Single, linear, qualitative AOPs are likely to be of limited use for the purposes of risk assessment. Biochemical and biological pathways are complex and tightly regulated in space and time, and that complexity needs to be reflected in the next generation of AOPs. Important also is that the basic tenet of toxicology is that the dose makes the poison and there is consequently a pressing need to ensure that wherever possible AOPs reflect quantitative elements of pathways, and in particular the threshold required for a pathway to progress from one key event to the next. Linked with this is a clear need to integrate AOPs with considerations of exposure metrics and toxicokinetics such that they become tools to support risk assessment, rather than solely for hazard identification. In addition to assessing product safety there is growing interest in the utilisation of effects based tools (EBT) and methods to improve the risk assessment of effects related to the large number of substances that need to be evaluated. For example, under the auspices of the European Union (EU) Water Framework Directive, a number of EBTs, including those specifically designed to measure modes of action in surface water, are being evaluated. These EBTs are being considered as tools for definition of environmental quality standards for specific modes of action to provide more holistic water quality monitoring (EU 2016a, b).

The specific areas that will be considered here are, in no particular order of importance: (a) quantitative AOPs and the importance of thresholds, (b) non-linear branching AOPs and AOP networks, (c) right to left AOP development/reverse engineering AOPs, (d) the need for ontologies to exploit existing information, and (e) from exposure to outcome pathways: towards a unified paradigm for AOPs.

### Quantitative AOPs and the importance of thresholds

Although in principle, AOPs differ little from considerations of MOA, they provide a pragmatic framework for an improved understanding of the mechanistic bases for MOA, and for describing the sequence of biological events and perturbations that can result in a specific type of adverse health effect. However, AOPs are currently described in purely qualitative terms—they do not currently provide an understanding of quantitative relationships between the MIE, subsequent KEs, and the ultimate adverse outcome. The important, and as yet unmet, requirement is that AOPs reflect the thresholds required for progress between KEs in the pathway leading to toxicity.

An illustrative example is provided by consideration of one of the first, and most complete, AOPs—that for skin sensitisation (OECD 2012; MacKay et al. 2013). It is particularly relevant for this purpose because it is known that chemical allergens differ by up to five orders of magnitude with respect to their relative skin sensitising potency (Kimber et al. 2011), and this quantitative understanding is required for risk assessment (Basketter et al. 2014).

The AOP for skin sensitisation identifies the MIE as the covalent reaction of a chemical with a host protein. Subsequently a number of KEs are described, in the following order: (a) keratinocyte activation and skin inflammation, with associated altered gene expression, (b) activation of dendritic cells (DC) and the several important contributions they make to uptake, processing and transporting of antigen to lymph nodes draining the site of exposure, and (c) the activation and proliferation of allergen-responsive T cells in these lymph nodes. This signals the induction of skin sensitisation. If the sensitised individual is re-exposed to the same allergen, at either the same or a different skin site, then the previously expanded population of allergen-responsive T cells, created during the final KE above, will mount an accelerated and more aggressive secondary immune response, resulting in cutaneous inflammation (allergic contact dermatitis; ACD). In this example ACD is the organism-level adverse health effect, or adverse outcome.

It is known that the vigour of T cell proliferation induced in lymph nodes draining the site of exposure to a chemical allergen correlates with the extent of sensitisation achieved (Kimber et al. 2011). However, what remain uncertain are the quantitative relationships between the MIE and the following KE, and between the sequence of KEs. For instance, how much adduct formation is required during the MIE to trigger the subsequent events in the skin that are required for the development of sensitisation? Is there a certain minimum (threshold) level of DC migration to lymph nodes that is necessary to support sufficient T cell activation and proliferation? These are not easy questions to address, but they are critical if the aim is to develop paradigms for assessment of relative skin sensitising potency without recourse to traditional animal-based methods, and will both improve hazard characterisation and facilitate risk assessment.

In all areas there may be some data regarding the thresholds characterising key event relationships (KERs) that already exist, and may include information on dose–response relationships, and temporal concordance, between KEs. However, these data may simply not yet have been captured, or been made available in a readily accessible form. If supporting evidence for KEs and KERs was suitably annotated with dose and time information, then it is possible that the temporal and dose-concordance between KEs could be inferred computationally based on Bradford Hill considerations (Becker et al. 2015).

### Non-linear/branching AOPs and AOP networks

The key principles for AOP development proposed by Villeneuve et al. (2014) acknowledge that biological pathways, including those perturbed as a result of toxicity, do not operate in isolation from each other, but suggest that it is most pragmatic to consider AOPs as single chains of KEs, each arising from one MIE and leading to one adverse outcome. As increasing numbers of AOPs are populated in the AOP-Wiki, common KEs involved in multiple AOPs are being identified. This allows information that has been curated for one AOP to be reused in another, avoiding duplication of effort. This does, however, indicate that it will become increasingly important to move away from viewing single linear AOPs in isolation, and to instead consider non-linear and branching AOPs within a broader context of AOP networks, as guidance is now acknowledging (OECD 2018). Combining multiple pathways, linking a single MIE with multiple biological outcomes, resulting in more realistic AOPs will bring important benefits informing future research, predictive and regulatory toxicology, and the development of non-animal methods (Jacobs et al. 2016). However, it is necessary to bear in mind that the conditions that propagate an AOP (i.e., causality) may differ depending on the specific circumstances (for example, according to animal species, cell type and exposure, etc.). It is, therefore, important that these and other relevant conditions are clearly defined and accurately recorded.

As discussed later in this article (see ontologies), existing information and descriptions of AOPs, KEs and KERs, will need to be consistent to enable the developments described above. In this context, it is relevant that the AOP-Wiki has recently been upgraded to facilitate the use of a defined set of ontologies to define ‘event components’ as consistent structured descriptions of KEs and their contexts (Ives et al. 2017).

### Right to left AOP development/reverse engineering AOPs

The current paradigm of the linear AOP encourages pathway development from left to right—MIE to adverse outcome. Thus, description of an AOP normally originates with identification of a MIE and then progresses sequentially through a number of KEs reflecting increasing levels of biological organisation and complexity until an adverse health or population effect is reached at the tissue or organism (or population) level (Ankley et al. 2010; Vinken 2013).

Although this approach is logical in terms of a progression from the earliest pivotal interaction of a toxicant with a biological system to the relevant adverse outcome, it does require some certainty, or at least understanding, of what the MIE and (at least some of) the KEs are, and the relationship between them. However, this is not always the case, and in such circumstances it may be informative to consider tackling construction of an AOP from the other end of the pipeline. That is, to work backwards from the adverse outcome of interest along the pathway of KEs (Perkins et al. 2011; Villeneuve et al. 2014).

Such an approach has been described in developing an AOP for chemical respiratory allergy (Kimber et al. 2014). It is well established that certain chemical allergens cause sensitisation of the respiratory tract resulting in allergic asthma and rhinitis. Although something is known of the relevant immunological and biological events, there remains uncertainty, and a lack of a scientific consensus, regarding the KE required for the acquisition of sensitisation. Specifically, there is a continuing debate about the role of IgE antibody, and the relative importance of other immunological effector mechanisms, in driving allergic sensitisation. The reverse engineered AOP for chemical respiratory allergy acknowledged this uncertainty and attempted to identify common ground that could provide a basis for the design of novel approaches for hazard identification and characterisation.

The learning that came from this is that in certain circumstances, where data are incomplete, or where there is uncertainty about a key element of the pathway leading to an adverse health effect; there may be merit in considering a reverse engineering approach.

### The need for ontologies to exploit existing information

The use of standard ontologies (that is, the formal naming and definition of terminology) and descriptions will enable more effective sharing of information contained within AOPs, particularly across different sectors. Good Laboratory Practice (GLP) animal studies may be reportable according to SEND (the Standard for Exchange of Non-clinical Data) for pharmaceuticals. Also, there is OECD guidance on in vitro test method reporting for both guideline (OECD 2005), and non-guideline methods (OECD 2014), and draft guidance for draft good cell culture practise (OECD 2017). However, although databases and data-sharing initiatives do exist, a lack of interoperable standards means there remain logistical and technological barriers to the optimal exploitation of data (Burgoon 2017; Ives et al. 2017). This could be addressed through greater consolidation and use of ontologies, and the accompanying development of improved bioinformatics and data mining tools; which in turn would drive greater use of available data to better define KERs and potentially allow automated linking of AOPs.

Opportunities exist to leverage existing ontologies and to agree universal standards—or a common language—for AOPs so that knowledge can be described and captured in a simple, sustainable way. Capitalising on the outcomes of existing consortia and data-sharing initiatives, and standardising ontologies used in publications will not only broaden the chemical space accessible from searches, but also reveal commonalities of underlying toxicities and biological pathways. Overcoming these practical and intellectual barriers will help to corroborate information in existing AOPs and also offer the prospect of more rigour in defining future AOPs.

Moreover, it is not just databases and data-sharing initiatives that hold information crucial for the effective development of AOPs; there is a wealth of useful information held in the basic scientific literature. AOPs are currently developed through the, largely manual, curation and integration of experimental data from the published literature on the biological effects of exposure to various xenobiotics, but computational approaches are being explored (Bell et al. 2016). With improved use of ontologies, integration of data contained within the existing scientific literature could potentially be automated and enhance computational integration to infer and propose AOPs, thereby accelerating their development.

### Exposure to outcome pathways: towards a unified paradigm for AOPs

A key requirement for furthering the effective use of AOPs is a clear understanding of the potential for human or environmental exposure to the chemical of interest. The application of AOPs to the development of risk assessments requires that an appreciation of the biological events and processes that might flow from encounter with a chemical is coupled with information about the conditions of exposure (including duration, route and extent), and fate of that chemical (Teeguarden et al. 2016).

As discussed above, an important aspect of AOPs is that there is a clear understanding of requirements (including levels of exposure in relation to target availability and dynamics) for the MIE to be triggered. The same is necessarily true for eliciting subsequent KEs in the AOP. There is, therefore, a need to extrapolate quantitative information on the relationships between tipping points for KEs in AOPs (where a biological system moves from an adaptive to an adverse response), in order that the predictions made can be considered in the context of realistic levels of human or environmental exposure. A continuing challenge will be to align an increasing sophistication of AOPs, and their more widespread application, with dosimetry and exposure metrics. How best to achieve this is still the subject of debate. However, there is a growing consensus that AOPs will best serve toxicology if methods can be found to integrate an appreciation of dose–response relationships and toxicokinetics with AOPs to build the concept of ‘source to outcome pathways’ that seek to embrace the totality of the processes involved in an aggregate exposure pathway (AEP) (Teeguarden et al. 2016; Vinken and Blaauboer 2016; Hines et al. 2018). Such a development will be of critical importance in consolidating AOPs as tools for risk assessment as well as hazard identification.

## Conclusions

It is already clear that AOPs are a force for good in toxicology. They have created a new lexicon for investigating and describing mechanisms and modes of action, they have proven useful for considering new targets and new paradigms for hazard identification and characterisation, and they continue to provide exciting opportunities for the design and implementation of non-animal approaches in toxicology and application of the 3Rs (reduction, refinement and replacement) principles.

Much has been achieved already, but it is timely now to consider the future trajectories of AOPs to ensure that their potential utility in toxicology is fully realised, and importantly, that they evolve into tools that will support the development of new and improved approaches to risk assessment. In this short article, we have highlighted what, in the view of these authors, are some of the opportunities that are now available to create a second generation of AOPs. The areas identified are not exhaustive, nor are they intended to be; doubtless there are other improvements and modifications that will also bring benefits, and one purpose of this short article is to stimulate further discussion about how best to exploit fully the opportunities provided by AOPs.
